# Tissue distribution of ethanol after intraprostatic injection using a porous needle

**DOI:** 10.3389/fonc.2022.1063781

**Published:** 2023-01-04

**Authors:** Megan N. Eubank, Ján Švihra, Kevin C. DiBona, Matthew Sommers, Tyler Oe, Ján Strnádel, Juraj Miklušica, Peter Szépe, Juraj Marcinek, Benjamin J. King, Mark K. Plante, Ján Ľupták, Mads Hvid Aaberg Poulsen, Masatoshi Kida, Eduard Baco, Ján Švihra, Peter Zvara

**Affiliations:** ^1^ Biomedical Laboratory and Research Unit of Urology, Department of Clinical Research, University of Southern Denmark, Odense, Denmark; ^2^ Department of Surgery, Larner College of Medicine, University of Vermont, Burlington, VT, United States; ^3^ Department of Urology, Jessenius Faculty of Medicine in Martin, Comenius University in Bratislava, Martin, Slovakia; ^4^ Department of Ophthalmology, University of Pittsburgh, Pittsburgh, PA, United States; ^5^ General Surgery, Atlantic Health System, Morristown, NJ, United States; ^6^ Biomedical Center, Jessenius Faculty of Medicine in Martin, Comenius University in Bratislava, Martin, Slovakia; ^7^ Department of Surgery and Transplant Center, Jessenius Faculty of Medicine in Martin, Comenius University in Bratislava, Martin, Slovakia; ^8^ Department of Pathological Anatomy, University Hospital Martin, Martin, Slovakia; ^9^ Research Unit of Urology, Department of Clinical Research, University of Southern Denmark, Odense, Denmark; ^10^ Department of Urology, Odense University Hospital, Odense, Denmark; ^11^ Department of Pathology and Laboratory Medicine, Larner College of Medicine, University of Vermont, Burlington VT, United States; ^12^ Department of Urology, Oslo University Hospital, Oslo, Norway

**Keywords:** Ultrasound-guided targeted injection, porous needle, tissue diffusion, prostate, organ-confined prostate cancer

## Abstract

**Purpose:**

To develop a safe and precise method for intraprostatic injection, and to establish correlation between the volume of ethanol injectate and the volume of subsequent infiltrated prostate tissue.

**Materials and methods:**

We performed intraprostatic injection of 96% ethanol using a needle which has a segment of its wall made of capillary membrane with hundreds of pores in an acute and chronic canine experiment, in heart-beating cadaveric organ donors, and in a xenograft model of human prostate cancer. Whole mount tissue sections were used for three-dimensional reconstruction of the necrotic lesions and calculation of their volumes.

**Results:**

The ethanol injection resulted in oval shaped lesions of well-delineated coagulative necrosis. In both healthy human and canine prostates, the prostatic pseudocapsule and neurovascular bundle remained intact without evidence of disruption. There was a linear correlation between administered volume of ethanol and the volume of necrotic lesion. Regression analysis showed strong correlation in the acute canine experiments and in experiments performed on xenografts of human prostate cancer. A formula was calculated for each experiment to estimate the relationship between the injected volume and the volume of infiltrated prostate tissue area.

**Conclusions:**

Intraprostatic injection using a porous needle allows for effective and predictable tissue distribution of the injectate in the prostate. Through varying the volume of the agent injected and use of needles with a different length of the porous segment, the volume of infiltrated tissue could be adjusted allowing for targeted focal treatment.

## 1 Introduction

Over the last decade, focal therapies of organ-confined prostate cancer (PCa) have been developed with a goal of eradicating the disease, while limiting side effects and complications associated with whole organ therapies. The use of focal therapies is supported by high precision of multiparametric magnetic resonance imaging (mpMRI) in localizing clinically significant PCa (1, 2). Furthermore, it has recently been proposed that such nonmorbid treatment modalities, with negligible effects on urinary, sexual and bowel function, will be beneficial as an alternative to PCa surveillance, halting disease progression, and delaying or eliminating the need for active therapy (3, 4). Provided that targeted intraprostatic therapeutics become available over subsequent years, then intraprostatic injection may serve as an ideal delivery modality. Currently available prostate imaging allows for precise targeted intraprostatic needle deployment; however, a method for effective and predictable diffusion of the injectant has not yet been developed (5).

We have previously demonstrated that the prostatic pseudocapsule, acts as a barrier to the diffusion of injected 96% ethanol (EtOH), thus presenting an advantage over temperature-based focal treatment options (6). We have also documented that in contrast to a standard hollow core needle with a single opening at the tip, intraprostatic injection using a needle with a porous wall allows for diffusion of EtOH along the entirety of the 1 - 3 cm long porous segment, improving injectant distribution (7, 8). These findings provide a strong foundation for further exploration of the porous needle for intraprostatic delivery of PCa therapy. The goal of this study was to correlate the volume of EtOH injected and the size of necrotic lesion.

## 2 Materials and methods

All study procedures were approved by The Animal Experiments Inspectorate of the Danish Veterinary and Food Administration (approval protocol number 2018-15-0201-01497). All animals were treated humanely according to the Declaration of Helsinki. Institutional Review Board of the Jessenius University, Martin, Slovakia approved the studies, which involved cadaveric organ donors (approval protocol number EK 146/2018).

### 2.1 Animals and anesthesia

Male HsdHre Beagle dogs were purchased at one to two years of age from Envigo (Venray, NL). Seven-day acclimation period was observed after arrival. Morning of the surgery the animal was premedicated with medetomidin (0.004 mg/kg), metadon 0.3 mg/kg and acepromazil 0.02 mg/kg im, and subsequently anesthetized with propofol (1.5 – 2 mg/kg iv). The anesthesia was maintained using continuous infusion of propofol (20 mg/kg/hour) and fentanyl (12 µg/kg/hour). Bolus of pentobarbital 140 mg/kg was given as a means of euthanasia. Paracetamol (10 mg/kg) was administered in iv infusion over 15 minutes at the conclusion of surgery in the chronic experiments.

### 2.2 The microporous needle

**Figure 1:**
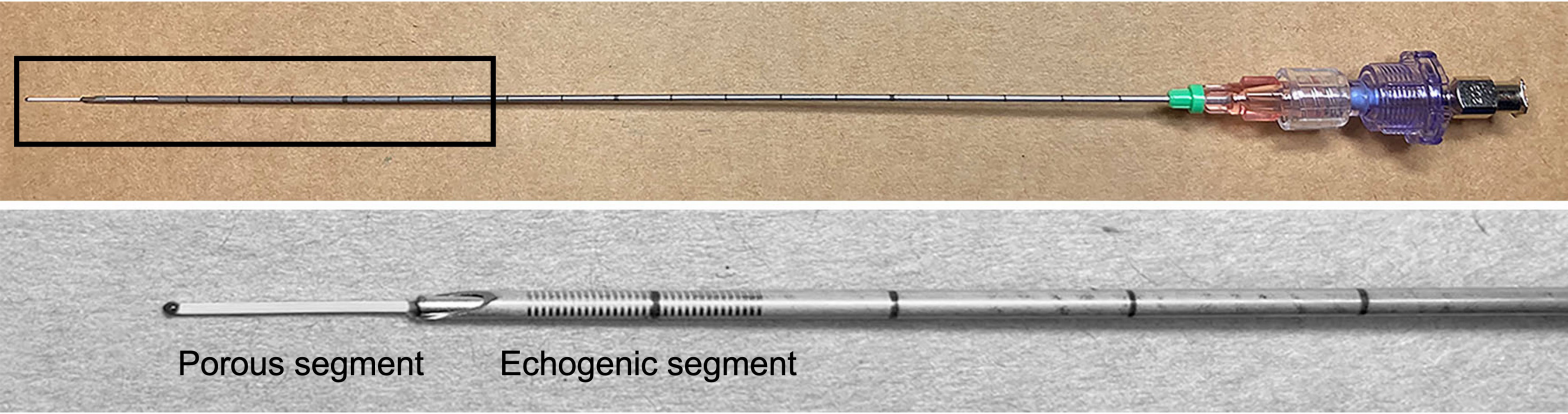
The microporous needle comprises a 20 Gauge cannula body with distal 1 cm porous segment. Included with the cannula is a stabilizing sheath for introducing the microporous segment into tissue target. Sheath has an echogenic sharp tip, which allows for accurate deployment of the microporous segment into the targeted tissue site under the ultrasound control. **Top:** Once device is in position, the sheath is retracted exposing the porous segment for injection. **Bottom:** High power magnification of the distal portion of the needle with the retracted stabilizing sheath.

In contrast to the classic needle with an opening on the tip, injection using a microporous needle takes place along the length of the porous segment, which contains hundreds of pores with maximal pore size 0.5 µm. The microporous needle is deployed into the tissue protected by an 18 Ga (1.27 mm) sheath. The tip of the sheath is sharp and made of echogenic material to facilitate placement of the microporous segment of the needle accurately into the target tissue under the ultrasound control **Figure 1**.

### 2.3 Acute canine experiment

**Figure 2:**
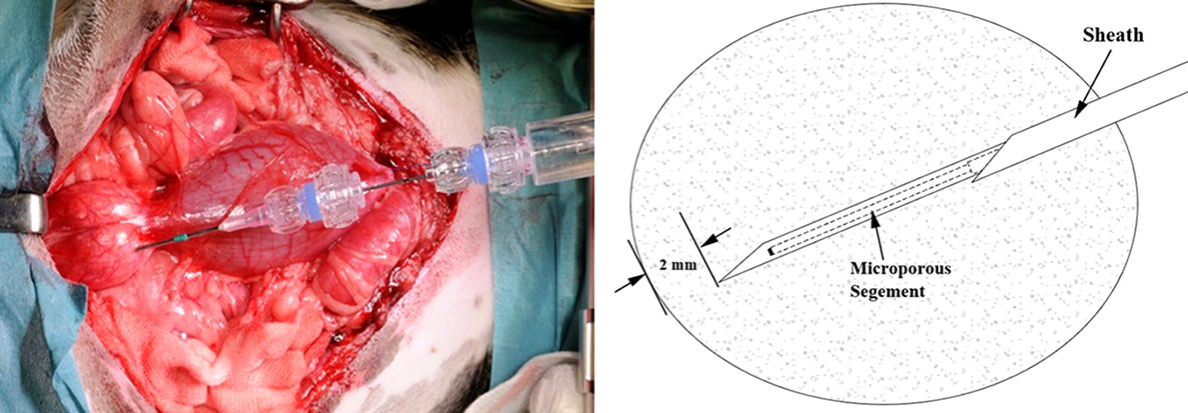
**Left.** Intraoperative image of the porous needle attached to the fixation apparatus and deployed into the left lobe of the prostate. **Right.** Schematic drawing showing the sharp tip of the protective sheath with the porous segment of the needle inside it. After the proper position of the needle is achieved, sheet is retracted to expose the porous segment.

Intraprostatic EtOH injections were performed in 8 beagle dogs. Under general anesthesia, prostate exposure was performed using lower midline abdominal incision. The porous needle was stabilized with a fixation apparatus attached to the operating table and was deployed into the lateral lobe of the prostate. Once the needle was in position, the porous segment was exposed by retracting the needle sheath. The tip of the needle and the sheath were kept inside the prostate, at least 2 mm from the prostatic pseudocapsule (**Figure 2**). EtOH was injected into the prostate at a rate of 15 mL/hour using an infusion pump. In the dose finding experiment, we administered 2 and 3 mL of EtOH into the left and right lobe of the prostate, respectively, to determine the maximal possible dose. In the remaining experiments, we injected 0.5, 1, 1.5, or 2 mL into each prostatic lobe. We used a needle with a 1 cm long microporous segment in 5 dogs, while a 2 cm long segment was used in one experiment. The tissue resistance during injections was measured by including the pressure transduced between the pump and the needle. To avoid extraprostatic leak, the needle was left deployed in the prostate for an additional two minutes after the predetermined volume of EtOH was administered and infusion pressure returned to baseline. Two hours following the second injection, while still under general anesthesia, prostates were resected and periprostatic area was carefully inspected for signs of extraprostatic EtOH effects.

### 2.4 Chronic canine experiment

In the chronic experiments, intraprostatic injections of 0.5, 1, 1.5, and 2 mL of EtOH, using the procedure described in the acute experiment, were performed using needles with a 1 cm long porous segment on eight dogs. The laparotomy was closed in two layers. The blood alcohol levels were measured from a sample taken from the jugular vein 20 minutes after the second intraprostatic EtOH injection. The animals were closely observed. During the first 24 hours post-op, animals were examined in 4 – 5-hour intervals for symptoms of pain, distress, fever, blood in stool and urine, and for problems with voiding. Daily observations were carried out throughout the entire postoperative period. Animals were weighed every 4 days. Any deviation from normal was recorded. The animals were euthanized 4 weeks after injection, prostates were removed and inspections of periprostatic areas were performed.

### 2.5 Intraprostatic injection in heart-beating cadaveric organ donors

Prostates of heart-beating cadaveric organ donors (HBCD) were injected with EtOH transperineally under real time visualization with three-dimensional transrectal ultrasound (Koelis, Meylan, France). With the patient in standard lithotomy position, the perineal skin was cleaned with 70% EtOH and betadine. An ultrasound probe was inserted into the rectum and secured in place using a probe holder. Subsequently, the needle was guided into the prostate using a grid attached to the ultrasound probe. The porous section of the needle, protected by a sheath, was deployed into the center of the lateral lobe of the prostate. The EtOH was delivered at a rate of 15 mL/hour. We performed injections in both lateral lobes, administering either 2 or 3 mL of EtOH per prostate lobe. The needle was left deployed in the prostate for an additional two minutes after the predetermined volume of EtOH was administered. The prostate was harvested a minimum of 6 (6–16) hours following the second injection, after all organs for transplantation were harvested.

### 2.6 Injection into the human PCa xenograft in a mouse

In order to study the ethanol diffusion in the PCa tissue, we used a mouse model of human PCa. The PCa cell line PC-3 (ATCC, Rockville, MD) and cultured in F-12K medium (Cat.No.: ATCC 30-2004), supplemented with heat-inactivated fetal bovine serum (10%, Gibco) and Penicillin/Streptomycin (1%, ThermoFisher Scientific). Before xenografting, cells were trypsinized and the pellet was dissolved in a 1:1 solution of medium and matrigel. 5 x 10^6^ cells were injected subcutaneously using an insulin syringe into the back of athymic mice (Crl : NU(NCr)-FoxN1Nu, age-7 weeks). Animals were observed and once the tumor reached 1.2 cm in diameter, ethanol injections were performed. A one centimeter long microporous cannula was introduced into the center of the PCa xenograft on the animal’s back. Due to the small tumor size, but easy accessibility right under the skin, the injection was performed manually by two investigators, without the use of a fixation apparatus. One held the tumor and guided the needle, the other investigator performed the sheet retraction and was operating the infusion pump. A flow rate 15 mL/hour was used to inject a volume of 250, 500 or 1000 µL (according to the tumor size). Tumors were resected 2 hours following the injection and the volume of necrosis was determined.

### 2.7 Tissue processing and histological examination

The procedures used were described in detail previously (7). Briefly, whole prostates and human PCa tumors were fixed in 10% neutral buffered formaldehyde, then sectioned into 2.5 mm step sections, which were oriented parallel to the base of the prostate. Five µm thick whole mount sections were placed on glass slides and stained with hematoxylin and eosin. Histology slides were converted to digital images. Areas of necrosis were delineated, sections were stacked, and the lesions were reconstructed to determine volume with stereology software Stereo Investigator 9.14.5 (Microbrightfield Bioscience, Williston, Vermont).

### 2.8 Statistical methods

Calculated volumes of the necrotic lesion were expressed as average ± standard deviation. A regression analysis was used to infer relationships between the volume of ethanol injected and the size of the necrotic lesion. The coefficient of determination R^2^ > 0.75 signified high level of correlation. A formula was calculated to estimate the correlation between the administered EtOH volume and the volume of necrotic lesion.

## 3 Results

### 3.1 Acute canine study

EtOH infusion proved impossible in one of 8 animals due to small prostate size. No extraprostatic leaks during injection were noted in any of the remaining animals. The infusion pressure reached the maximum of 234 ± 36 mm Hg and remained stable throughout the injection.

### 3.2 Intraprostatic EtOH diffusion

**Figure 3:**
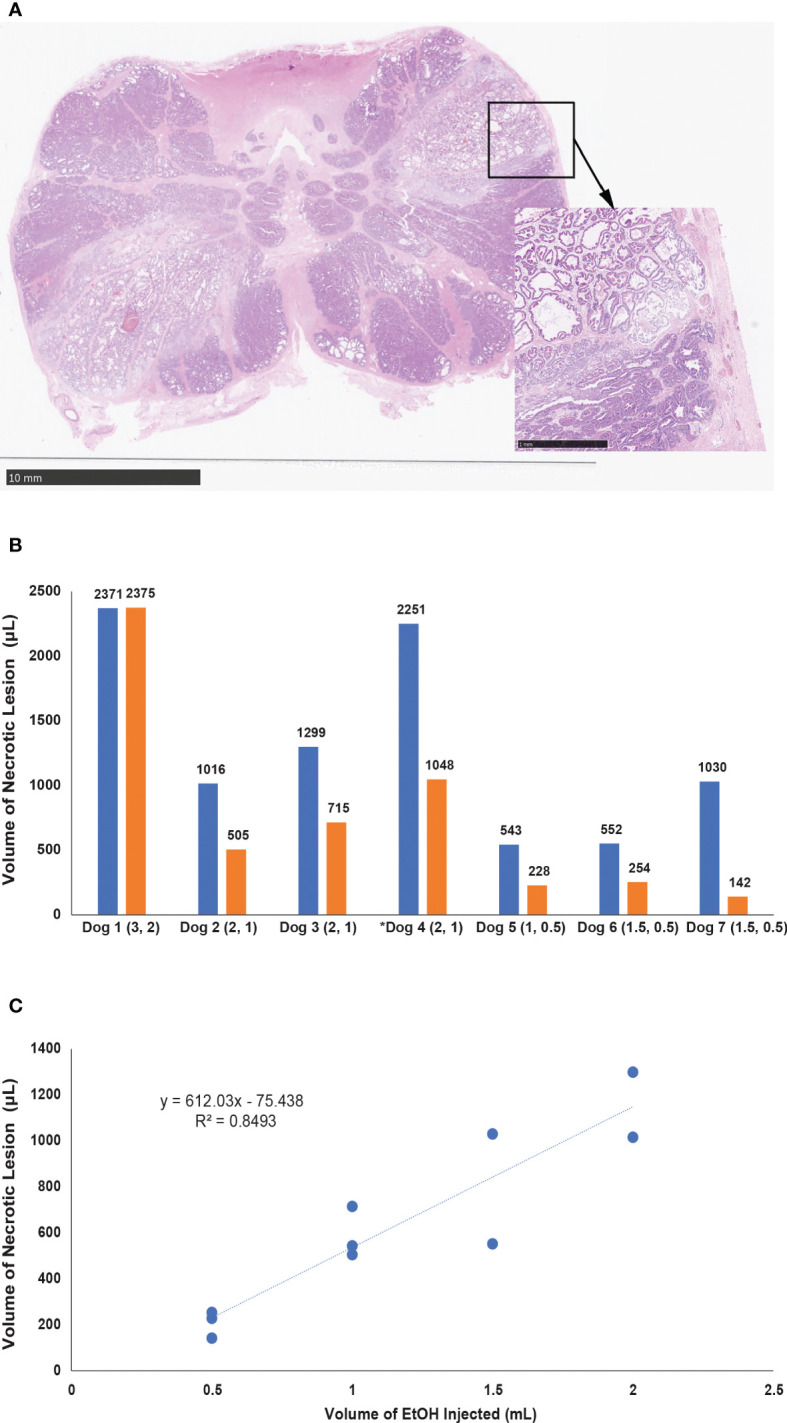
**(A)** Section of the canine prostate two hours after EtOH injection, oriented parallel to the prostate base. High-power magnification (marked by arrow) depicting intact fibromuscular pseudocapsule adjacent to the necrotic lesion. **(B)** Graph summarizing the volume of tissues lesions in 7 canine prostates 2 hours post injection. The volume injected (mL) is listed in brackets under x axis, number above each bar represents the volume of the lesion. Dog 1 was used in a dose finding experiment and the Dog 4 (marked with asterisk) represents injection with a 2 cm-long porous needle. **(C)** Plot demonstrating the correlation of the volume of ethanol and the volume of necrotic lesion in all experiments where needle with 1 cm long porous segment was used for injection.

On histological evaluation of the dose-finding experiment, we identified a necrotic lesion involving most of the prostate tissue. The two lesion sites in this dog overlapped, likely due to the high volume injected, preventing us from establishing the correlation between the volume of ethanol and volume of the necrotic lesion. In the remaining 6 prostates, EtOH diffused throughout the glandular tissue and smooth muscle, resulting in glandular dilatation, shrinkage of cell nuclei, and loss of visible nucleoli. EtOH volume of 0.5 – 2 mL caused well-delineated necrotic lesions. The prostatic pseudocapsule remained intact without evidence of disruption, even in cases when intraprostatic necrotic area developed adjacent to it (**Figures 3A**). We did not identify EtOH effects on the prostatic urethra and the structures surrounding the prostate.

### 3.3 Correlation between injected volume and size of the necrotic leasion

Injections of 0.5, 1.0, 1.5, and 2.0 mL with the 1 cm porous segment resulted in lesion volumes of 208 ± 58, 588 ± 112, 791 ± 338, and 1158 ± 200 µL, respectively (**Figures 3B**). A linear correlation between administered volume of EtOH and the volume lesion generated following formula: x = (y + 0.7443)/0.612, where x corresponds to the volume of administered EtOH and y corresponds to the volume of the lesion in mL. Regression analysis of the relationship between the volume of EtOH injected and the size of necrosis (R^2^ = 0.85) showed a high level of correlation (**Figure 3C**). The 2 cm microporous segment needle resulted in larger areas of tissue infiltration, 1048 µL for injection with 1 mL and 2251 µL for injection with 2mL.

### 3.4 Chronic canine study

**Figure 4:**
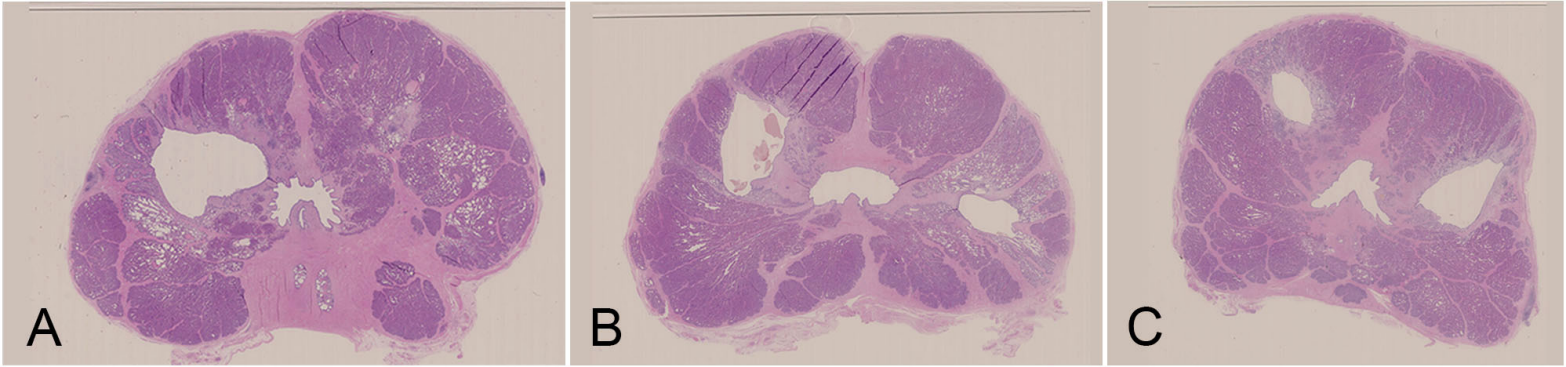
Tissue defects developed 4 weeks after intraprostatic injection of absolute ethanol. **(A)** 2 mL (left) and 0.5 mL (right), **(B)** 2 mL (left) and 1 mL (right), **(C)** 1.5 mL on both sides. Tissue defects are lined with epithelium and surrounded by connective tissue with signs of fibrosis. The area of the prostatic pseudocapsule shows no abnormalities.

The blood alcohol levels following intraprostatic injection ranged between 0.5 to 4.3 mmol/l. No immediate intra- or postoperative complications occurred. Two animals developed swelling of the wound and transient increase in body temperature to 38.5 and 39°C, which lasted less than 24 hours. None of the animals developed hematuria and no evidence of blood in the stool was detected. Prostatic urethra and all structures surrounding the prostate were intact. Hematoxylin and eosin-stained whole mount sections of the prostates showed tissue defects (cavities) in all prostatic lobes injected with 1, 1.5 and 2 mL (**Figure 4**). Tissue defects were lined with epithelium and surrounded by connective tissue cells typical of early fibrosis. The area of the prostatic pseudocapsule, consisting of loose connective tissue, showed no abnormalities.

### 3.5 Intraprostatic injection in HBCD

**Figure 5:**
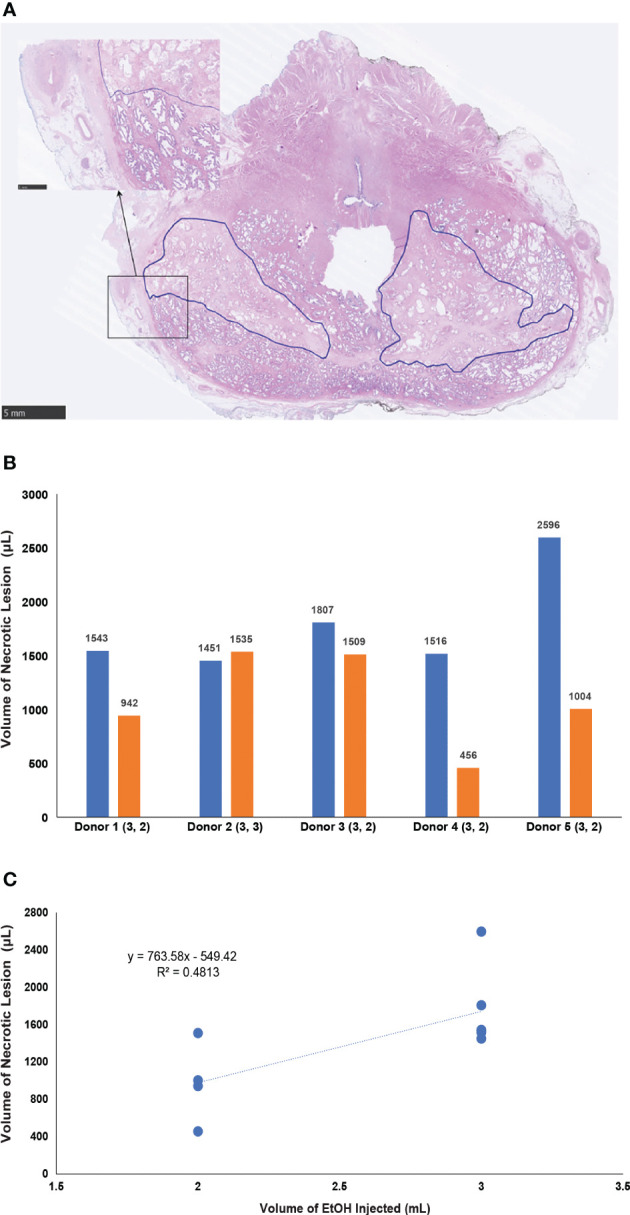
**(A)** Section of the human prostate 8 hours after transperineal intraprostatic ethanol injection. Necrotic areas (traced by blue line) have irregular borders. High power magnification marked by arrow, shows intact prostatic pseudocapsule. **(B)** Graph summarizing the volume of tissues lesions in 5 human prostates 8 - 16 hours post injection. The volume injected (mL) is listed in brackets under x axis, number above each bar represents the volume of the lesion. **(C)** Plot demonstrating the correlation of the volume of ethanol and the volume of necrotic lesion.

A total of 6 organ donors were included in the study. No extraprostatic leaks were noted during injection, and no post-injection complications were documented during close observation in the intensive care unit. One prostate was excluded due to suboptimal tissue processing. On histological analysis of the remaining prostates, every EtOH injection resulted in formation of necrotic lesion. Most lesions were irregular in shape. Areas of necrosis demonstrated coagulative-type tissue changes with significant alteration of native histological architecture including shrinkage of cell nuclei and disruption of glandular columnar epithelium. The prostatic pseudocapsule remained intact, even in cases where the lesion was positioned adjacent to it (**Figure 5A**). Injections of 2.0 mL and 3.0 mL with the porous section of the needle resulted in average lesion volumes of 980 ± 430 and 1740 ± 440 µL, respectively (**Figure 5B**). A linear correlation between injected EtOH volume and lesion volume generated the following formula: x = (y + 0.5488)/0.7634, where x corresponds to the volume of EtOH and y corresponds to the volume of necrotic lesion in µL. Regression analysis (R^2^ = 0.48) demonstrated moderate level of correlation (**Figure 5C**).

### 3.6 Injection into the human PCa xenograft in a mouse

**Figure 6:**
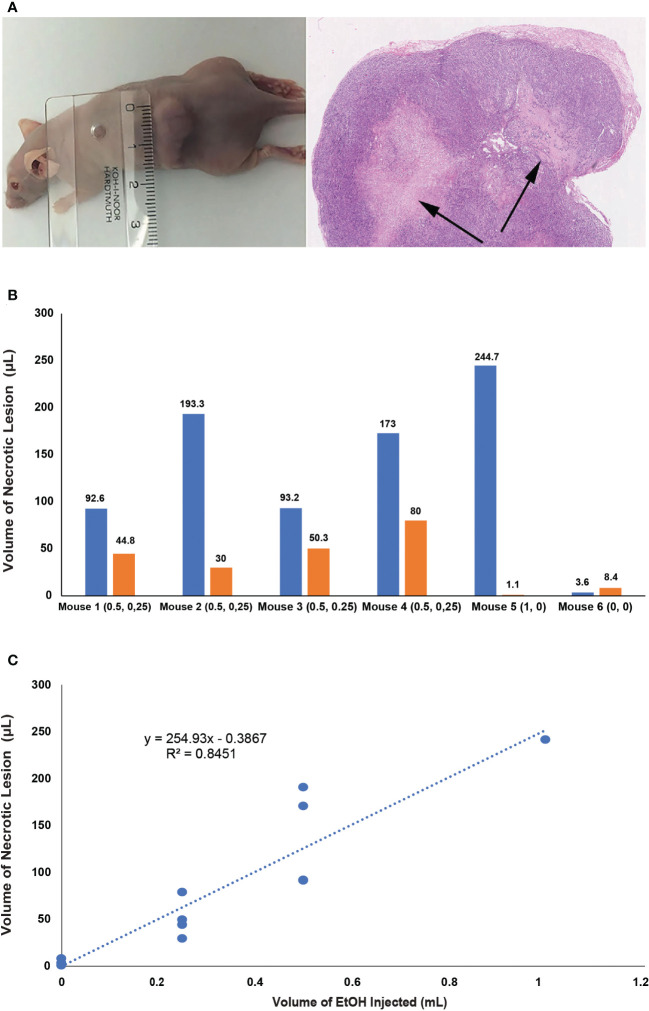
**(A)**Left. The xenograft of human prostate cancer localized subcutaneously in the back of athymic mice. **Right.** Section of the prostate cancer tissue two hours after EtOH injection. Necrotic lesions are marked by arrows. **(B)** Graph summarizing the volume of tissues lesions in human prostate cancer xenografts 2 hours post injection. The volume injected (mL) is listed in brackets under x axis, number above each bar represents the volume of the lesion. **(C)** Plot demonstrating the correlation of the volume of ethanol and the volume of necrotic lesion in 3 controls and 9 experiments where ethanol was injected trough 1 cm long porous segment.

The total number of prostate cancer tumors was 12. Ethanol was instilled into 9 tumors. Three tumors were used as controls. Injections of 0, 250, 500 and 1000 µL with the porous section of the needle resulted in average lesion volumes of 4.4 ± 3.0, 52.3 ± 18.2, 138.0 ± 45.7 and 244.7 µL respectively. Regression analysis (R^2^ = 0.85) demonstrated strong level of correlation (**Figure 6**).

## 4 Discussion

Prostate is well accessible for injection and new imaging modalities allow for delineation of the clinically significant PCa lesion and its precise targeting. We have previously demonstrated that prostatic pseudocapsule acts as a barrier to the diffusion of injected substance. If a precise method for intraprostatic injection is developed, a cytotoxic or a PCa-specific therapeutic agent could be injected in a manner that would allow for tumor infiltration while avoiding injury to extraprostatic anatomical structures and prostatic urethra. Up until now, use of intraprostatic injection in the treatment of prostate disease has been limited to early phase clinical trials of antiandrogen or gene therapy (3, 9). Intraprostatic injection of EtOH have been studied extensively for the therapy of benign prostatic hyperplasia (10–12). All clinical studies published to date have used standard hollow core needles with a single opening at the tip. In our previous research, we have documented that when the hollow core needle is used, tissue diffusion in the prostate is highly variable, based on the type of tissue the tip of the needle is embedded in. We also documented that backflow along the needle tract is a significant problem (6).

To address this issue, we used injection through a needle which had a segment of its wall made of capillary membrane with hundreds of pores 0.5 µm in size. Constant hydraulic force generated by the infusion pump resulted in homogenous distribution of the infused substance through the adjacent tissue area, with majority of the infusate infiltrating the interstitial space (13).

The primary goal of this study was to establish correlation between the volume of the drug and the infiltrated area of the tissue. In our previously studies, we used injection into a normal prostate of a dog (14). We aimed to continue these studies, but also examine tissue distribution in healthy human prostates and human PCa tissue. EtOH caused coagulative necrosis, which could be clearly delineated on whole mount sections of the prostate. In the canine prostate, 2 mL proved to be the highest volume which resulted in a circumscribed necrotic lesion limited to a single prostatic lobe. In the acute canine study, we documented a strong correlation between the volume of EtOH and size of the lesion. Moderate correlation was documented in subsequent study on HBCD. The lower degree of correlation was likely due to the larger size of human prostate glands which allowed for larger variability in EtOH tissue distribution. The high degree of correlation between the injected volume of EtOH and lesion size and high regularity of distribution was observed in the xenografts of PCa tissue.

The secondary goal of this study was to obtain data assessing the safety of the procedure. We did not identify disruption of the prostatic pseudocapsule in any of the prostates, even though several EtOH injections resulted in necrotic lesions that reached to the pseudocapsule. No extraprostatic tissue injury was documented on inspection during prostate resection. In the chronic canine study, the animals were closely monitored for signs of extraprostatic tissue injury, including injury to bowel or urinary bladder. The postoperative care and recording of symptoms was conducted by staff of the pre-clinical contract research organization (Citoxlab, A Charles River Company, Ejby, Denmark) where the chronic experiments were performed. Citoxlab staff provided their services but were not otherwise associated with the study, limiting the bias. No symptoms suggestive of extraprostatic tissue damage were documented.

The study in HBCD allowed us to determine the feasibility of the transperineal intraprostatic EtOH injection. In contrast to the study which used intraprostatic injection of a large volume (up to 20 mL) antiandrogen 2-hydroxyflutamide in patients with low- or intermediate risk organ-confined PCa, the method we used involved small (≤ 3 mL) injectant volumes with the goal of infiltrating clinically significant lesions identified using mpMRI and targeted for drug delivery using the MRI-TRUS fusion.

The ultimate focus of the proposed targeted intraprostatic injection is treatment of organ-confined PCa. Therefore, a necessary step in the evaluation of this method is its implementation and further testing in patients suffering from organ confined PCa. We believe that if successfully developed, the targeted intraprostatic injection would allow for tailoring the treatment to individual patients. Through varying the volume of the injected agent and use of needles with different lengths of porous segment, intraprostatic injection could be adjusted to the size of the lesion. Different needle deployment method (transperineal or transrectal) could be used based on the location of the PCa lesion.

This study proposes a new method that would allow for effective and predictable tissue distribution of injectate in the prostate. In the acute experiments, we documented well-delineated areas of coagulative necrosis and a high degree of correlation between the injected volume of EtOH and lesion size in both healthy canine prostates and human PCa tissue. Four weeks post injection, ablation of prostate tissue was documented. No disruption to the prostatic pseudocapsule or evidence of injury to the periprostatic anatomical structures were observed. With the development of PCa therapeutics, alternative targeted therapies could be delivered using this injection protocol, achieving enhanced outcomes and preventing debilitating complications.

## Data availability statement

The raw data supporting the conclusions of this article will be made available by the authors, without undue reservation.

## Ethics statement

The studies involving human participants were reviewed and approved by Institutional Review Board of the Jesenius University, Martin, Slovakia. Written informed consent for participation was not required for this study in accordance with the national legislation and the institutional requirements. The animal study was reviewed and approved by The Animal Experiments Inspectorate of the Danish Veterinary and Food Administration.

## Author contributions

Conceptualization PZ, MP, JS, JM, JMa, JL, EB. Methodology PZ, ME, JSt, JSj, KD, MS, TO, PS, BK. Formal analysis ME, JSj, KD, MS, TO, MK, BK. Original draft preparation PZ, MS, BK. Review and editing BK, ME, KD. All authors contributed to the article and approved the submitted version.
